# Gold nanoparticles synthesis to application as nano biosensors

**DOI:** 10.1186/1753-6561-8-S4-P249

**Published:** 2014-10-01

**Authors:** Jairo Pinto Oliveira, Adilson Ribeiro Prado, Bárbara Altoé Milaneze, Wanderson Juvêncio Keijok, Dominik Lenz, Josimar Ribeiro, Moises Renato Nunes Ribeiro, Maria José Pontes, Breno Valentim Nogueira, Marco Cesar Cunegundes Guimaraes

**Affiliations:** 1 Universidade Federal do Espírito Santo (UFES), Vítoria, ES, Brazil; 2 Universidade Vila Velha (UVV), Vila Velha, ES, Brazil

## Background

The particularities of gold nanoparticles (AuNP's) have stimulated different areas of research in recent years. The exploitation of the optical properties, electronic and magnetic materials has allowed these his job in different fields of application, such as the construction of biosensors in gradual release system for drugs, lubricants, solar cells, catalysis and other [1].

The properties of the nanoparticles are dependent on the size and shape of the particles. The same material composition can be determined with different physical and chemical characteristics just modifying characteristics such as size, self-organization, crystalline structure and shape, the point of the material in the nanometer range show distinct physical and chemical properties of materials on the macroscopic scale [2].

The aim of this work was to optimize the synthesis of gold nanoparticles using the reduction method sodium citrate, assessing concentrations of this reducing agent and the synthesis time. The attempt is to develop a route to generate nanoparticles from a precursor and facilitate the use of these nanomaterials in biosensors.

## Methods

To check the effect of variables on conversion of the reaction, as well as finding the conditions that maximized the synthesis of nanoparticles, one factorial design (3^2^) with 3 levels and 2 variables was performed. The variables evaluated were: Concentration of Reducing Agent (1.0, 2.0 and 3.0%) and the time synthesis (5, 10 and 15 min). These intervals were defined to cover most of the studies described in the literature [3].

To prepare the AuNP's, the reducing agent (sodium citrate - Na_3_C_6_H_5_O_7_) was added under experimental design to the precursor solution gold (HAuCl_4 _2.5 × 10^-4 ^M) at 65°C and was maintained under stirring for the time evaluated.

AuNP's samples were collected after the synthesis step and had their optical properties assessed by spectrophotometry UV-visible (SHIMADZU). The size and morphology of AuNP's were examined by transmission electron microscopy (JEM-1400, JEOL Inc, USA).

## Results and conclusions

Results were obtained from the spectra of the solutions in electronics and suspension compared with the images of transmission electron microscopy, which showed the difference in particle size.

Surface charts were generated in order to optimize the results and Pareto was introduced to allow better visualization and identification of variables and interactions that affect the synthesis of gold nanoparticles (p <0,05).

The two variables were significant in the process and higher absorbance were found in greater synthesis times. It was concluded that the reducing agent acts as a stabilizer evaluated, since in higher concentrations there is a decrease in absorbance and thus, the smaller the size of the nanoparticles.
